# Reaching Frontline Providers With Updated HIV Clinical Guidance at Scale Under Declining Training Support: WhatsApp Microlearning During Rollout of 6‐Monthly ART Dispensing in South Africa

**DOI:** 10.1002/jia2.70222

**Published:** 2026-10-26

**Authors:** Briony Chisholm, Lynne Wilkinson

**Affiliations:** ^1^ Division of Clinical Pharmacology Department of Medicine University of Cape Town Cape Town South Africa; ^2^ International AIDS Society Johannesburg South Africa; ^3^ Centre for Infectious Epidemiology and Research University of Cape Town Cape Town South Africa

1

HIV management is guided by regularly updated clinical guidelines, and keeping frontline healthcare workers (HCWs) informed requires ongoing training. Traditional approaches—in‐person workshops/longer virtual sessions—are costly, slow to reach providers and disrupt clinical services. These challenges are growing as implementing partner (IP) support for facility‐level training declines due to global funding cuts.

In South Africa, with primary healthcare clinics spread across 1.2 million km^2^, in‐person training requires travel, accommodation and staff time away, while longer virtual sessions are often poorly attended due to duration, lack of protected time and limited promotion.

These challenges were acute for the phased rollout of facility‐based 6‐month multi‐month dispensing (6MMD) of antiretroviral therapy from August 2025. The timeline was short; virtual training was limited to a once‐off session for provincial stakeholders; and facility‐level IP support was reduced.

Previous HIV service delivery changes, such as repeat prescription collection strategies, took several years to reach national scale. One challenge was ensuring guideline updates reached facility‐level prescribers, with practical guidance on how to implement them. Rapid dissemination of revised guidance is, therefore, increasingly important.

Microlearning—bite‐sized units of learning [1]—offers a potentially cost‐effective, scalable and accessible solution. Delivered via mobile messaging, it avoids centralized gatherings and minimizes clinical service disruption. It is underpinned by cognitive load theory [1, 2] and has been used for continuing education across medical specialities [3, 4, 5, 6].

South Africa has near‐universal mobile phone access and widespread WhatsApp use [7]. WhatsApp Business, a free encrypted messaging platform, allows structured group communication and moderation. South African clinicians have used WhatsApp to discuss complicated HIV/TB cases and for continuing medical education [8]. A cluster randomized controlled trial in South African primary healthcare clinics found that WhatsApp‐based HIV microlearning significantly improved HCW knowledge and increased correct patient care by 21% in the year after training [9, 10].

The 6MMD microlearning curriculum was developed by a training consultant and a differentiated service delivery technical specialist, based on the updated 2025 national HIV guidelines and the content and questions arising from the National Department of Health (NDoH) virtual 6MMD training session. There was no formal co‐design process.

The developers grouped two or three related learning points into each of 8 weekly lessons. Each 10‐ to 15‐min session comprised approximately 20 sequential WhatsApp messages: text explanations, realistic case studies, polls requiring participants to apply guidance, explanatory feedback, one or two images and a summary voice note. The module was reviewed and approved by the NDoH.

The first training group served as a practical test; participant feedback and responses were used to refine wording, cases and explanations for subsequent groups.

Recruitment was led by provincial Department of Health (DoH) leads and IPs, and the training was introduced during the national virtual training session and on existing programme management platforms. A digital invitation, circulated nationally and provincially by DoH and partner stakeholders, linked via QR code to a SurveyMonkey registration form. WhatsApp groups are capped at 1024 members and participants cannot join once sessions have started, so additional groups were created on demand: three training groups started over a 2‐month period, with training running from August to November 2025.

The NDoH phased 6MMD implementation: Phase 1 (August 2025; 5 facilities/province); Phase 2 (September 2025; 10 facilities/province); and Phase 3 (April 2026; all facilities). Microlearning ran in parallel with Phases 1 and 2, focusing on these facilities but open to Phase 3 HCWs.

Sessions were held in the WhatsApp group at lunchtime, with low data requirements. Only admins could post, except briefly at the end of each session for participant questions. Participants could also contact trainers directly; questions of general relevance were shared with the group. Participants unavailable during sessions could read them—and vote in polls—when convenient.

A total of 2285 participants enrolled across the groups: 62.1% nurses, 10.8% doctors and 10.7% pharmacists; 58.7% were public sector and 27.7% partner organization staff. Participation was tracked using WhatsApp's “blue tick” function at three time points: within 1 h of the session (“live”), 24 h and 1 week. Blue ticks show that messages were opened, not that they were read or understood. Across all groups and sessions, 53% of participants viewed the session within 1 h, 82% within 24 h and 89% within 1 week (Figure 1). Live views fell from 67% in session 1 to 44%–57% in later sessions, while 1‐week views remained 85%–95%, highlighting the value of flexible access for busy providers.

**FIGURE 1:**
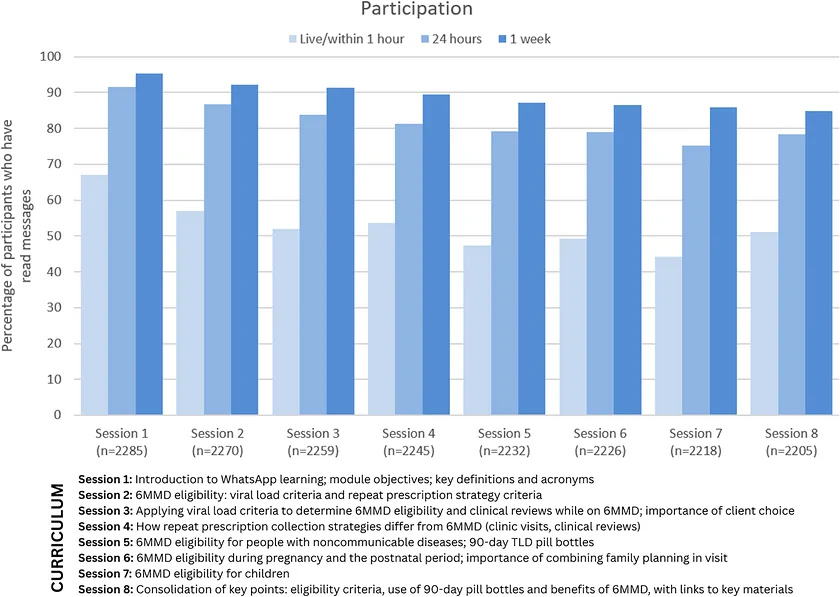
Participation in the eight sessions, measured by the “blue tick” function of WhatsApp at three time points after each session (three groups aggregated).

Polls administered after the final session had low participation: 294/2285 (12.9%) responded to the satisfaction questions; 79% (232/294) were highly satisfied and 20% (58/294) satisfied. Of 301 (13.2%) participants responding about future training, 99% (300/301) expressed interest. These findings are subject to substantial non‐response and selection bias and cannot be generalized to all participants.

An implementation poll 4 months after training, before decentralized training began, found that 78% (236/302) of respondents able to recommend/prescribe 6MMD or enrol eligible patients reported having done so. As participants included IP staff, trainers and pharmacists who did not perform these functions, and the number eligible to respond was not recorded, these results should be interpreted cautiously.

Several implementation lessons emerged:

1. Adapting longer virtual training into WhatsApp microlearning requires prioritizing a small number of learning points per session, applied through cases and polls and reinforced through text, visual and audio formats. The 6MMD module informed subsequent modules on tuberculosis preventive treatment and re‐engagement, strengthened by clinician review and external technical review by an independent third‐party organization before final NDoH approval.
2. Lesson development is more complex than it initially appears, requiring collaboration between technical experts and training designers and multiple rounds of revision to ensure clarity and fidelity to national guidelines.
3. Structured moderation is essential: the training consultant delivered the lesson with the technical specialist as moderator.
4. Rapid implementation is feasible with programme leadership support: only 6 weeks elapsed between the first planning discussion and the launch of the first training group.
5. Recruitment is the main operational challenge. Leveraging existing programme communication channels and professional WhatsApp networks proved critical, with intensive recruitment follow‐up in Phase 1/2 facilities.
6. A decentralized district‐level model was needed for broader HCW coverage. IPs were trained to set up district‐level groups and deliver the same module for Phase 3 in March 2026.
7. Training groups may also provide a mechanism for ongoing implementation support: follow‐up polling 4 months post‐training provided rapid feedback on barriers to 6MMD rollout.

This experience suggests that WhatsApp‐based microlearning, designed to minimize service disruption, can rapidly disseminate clinical guidance to large numbers of frontline HCWs. As resource constraints grow and IP support for facility‐level training declines, scalable digital approaches may complement existing training, maintain continuous professional education and support rapid implementation of guideline updates.

A full package of WhatsApp‐based microlearning implementation guidance has been developed and described elsewhere [11].

Future work will explore how to embed this approach within NDoH training on new and updated guidelines and using decentralized delivery through DoH training structures as part of a blended approach.

## Author Contributions

BC and LW conceptualized and developed the field note. Both authors have reviewed and approved the final manuscript.

## Funding

This work was supported through investments (INV‐047567 and INV‐094257) from the Gates Foundation to the IAS—International AIDS Society.

## Conflicts of Interest

The authors declare no conflicts of interest.

## Data Availability

The data that support the findings of this study are available from the corresponding author upon reasonable request.
